# Supplementary material for: Miniature spatial transcriptomics for studying parasite-endosymbiont relationships at the micro scale
Source: Nat Commun. 2023 Oct 14;14:6500. doi: 10.1038/s41467-023-42237-y (PMC10576761; doi:10.1038/s41467-023-42237-y)
Supplement: Supplementary file 1 — Supplementary Information [file 41467_2023_42237_MOESM1_ESM.pdf]

Supplementary Figures

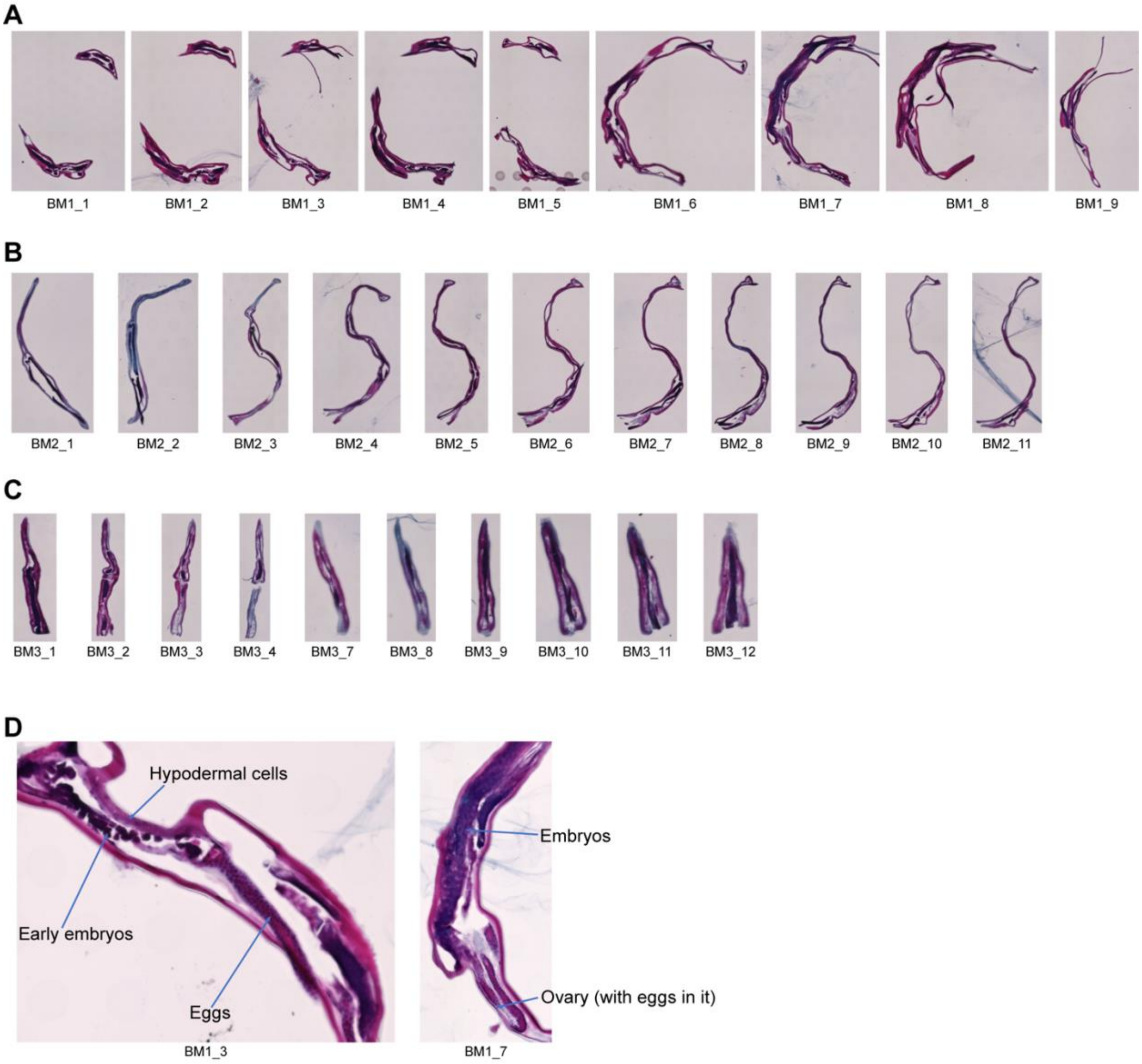

Supplementary Figure 1. H&E images and morphology of study tissue sections. H&E images of the tissue consecutive sections from worm samples BM1 (A), BM2 (B), and BM3 (C) used in the study. Section names are below each section image. (D) Zoom-in of tissue section BM1_3 and BM1_7 accompanied by morphological annotations. Magnification 20x.

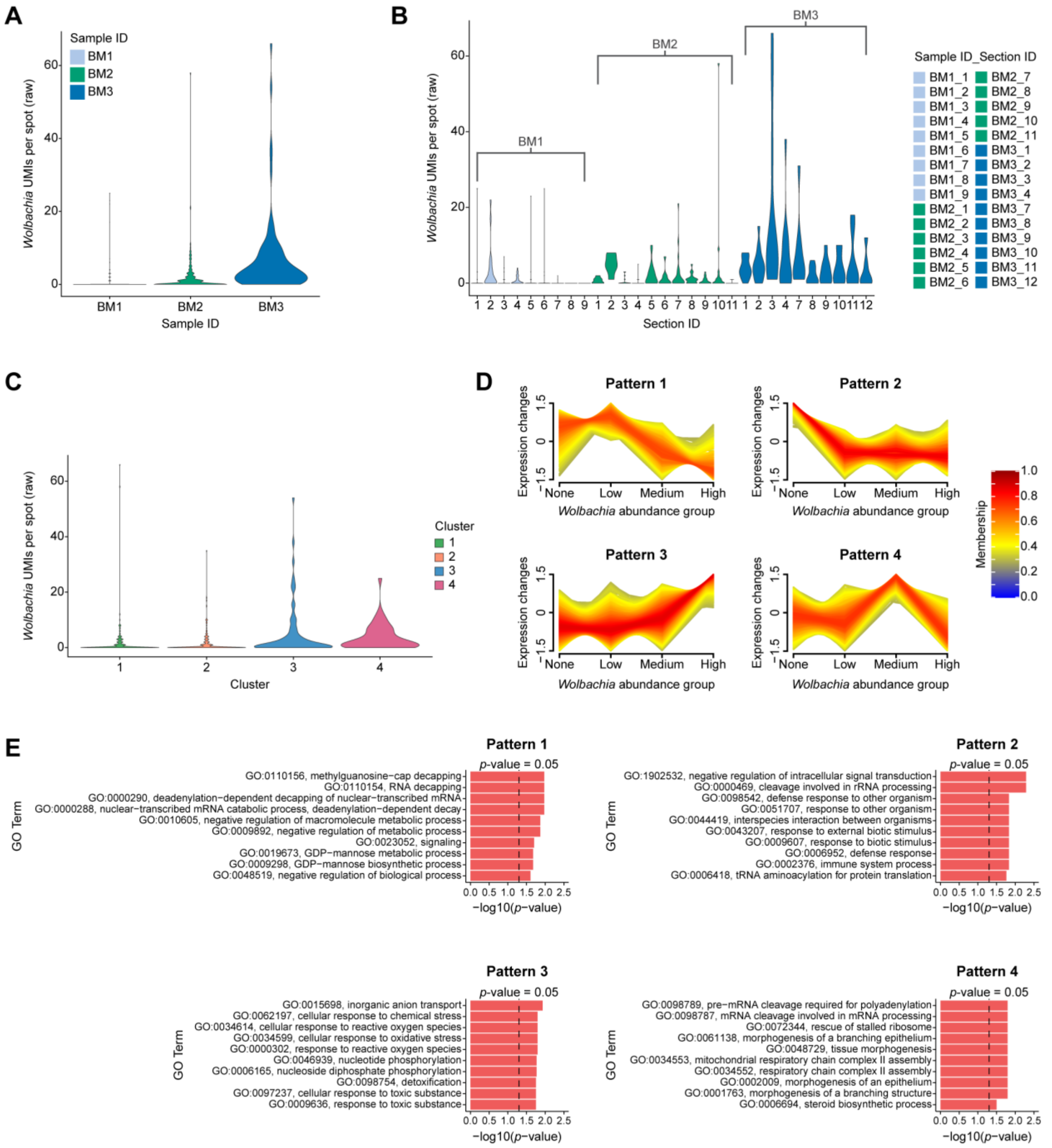
A
Sample ID
BM1
BM2
BM3
Wolbachia UMIs per spot (raw)
Sample ID
B
BM1
BM2
BM3
Section ID
Sample ID_Section ID
C
Cluster
Wolbachia UMIs per spot (raw)
D
Pattern 1
Pattern 2
Pattern 3
Pattern 4
Expression changes
None Low Medium High
Wolbachia abundance group
Membership
E
Pattern 1
p-value = 0.05
GO:0110156, methylguanosine−cap decapping
GO:0110154, RNA decapping
GO:0000290, deadenylation−dependent decapping of nuclear−transcribed mRNA
GO:0000288, nuclear−transcribed mRNA catabolic process, deadenylation−dependent decay
GO:0010605, negative regulation of macromolecule metabolic process
GO:0009892, negative regulation of metabolic process
GO:0023052, signaling
GO:0019673, GDP−mannose metabolic process
GO:0009298, GDP−mannose biosynthetic process
GO:0048519, negative regulation of biological process
GO Term
−log10(p−value)
Pattern 2
GO:1902532, negative regulation of intracellular signal transduction
GO:0000469, cleavage involved in rRNA processing
GO:0098542, defense response to other organism
GO:0051707, response to other organism
GO:0044419, interspecies interaction between organisms
GO:0043207, response to external biotic stimulus
GO:0009607, response to biotic stimulus
GO:0006952, defense response
GO:0002376, immune system process
GO:0006418, tRNA aminoacylation for protein translation
Pattern 3
GO:0015698, inorganic anion transport
GO:0062197, cellular response to chemical stress
GO:0034614, cellular response to reactive oxygen species
GO:0034599, cellular response to oxidative stress
GO:0000302, response to reactive oxygen species
GO:0046939, nucleotide phosphorylation
GO:0006165, nucleoside diphosphate phosphorylation
GO:0098754, detoxification
GO:0097237, cellular response to toxic substance
GO:0009636, response to toxic substance
Pattern 4
GO:0098789, pre−mRNA cleavage required for polyadenylation
GO:0098787, mRNA cleavage involved in mRNA processing
GO:0072344, rescue of stalled ribosome
GO:0061138, morphogenesis of a branching epithelium
GO:0048729, tissue morphogenesis
GO:0034553, mitochondrial respiratory chain complex II assembly
GO:0034552, respiratory chain complex II assembly
GO:0002009, morphogenesis of an epithelium
GO:0001763, morphogenesis of a branching structure
GO:0006694, steroid biosynthetic process

Supplementary Figure 2. ***B. malayi*** **gene expression correlation with** ***Wolbachia*** **abundance variation. A-C** Violin plots of the *Wolbachia* unique molecules (UMIs) per spot (raw) across the different worm samples used in the study (**A**), the different worm sample sections (sample name_consecutive section #) used in the study (**B**), and the *B. malayi* clusters (**C**). **D.** MFuzz soft clusters (Patterns 1-4) of *B. malayi* genes co-expressed across different *Wolbachia* abundance groups. **E.** Bar plots of unadjusted *p*-values (*p*) of the top enriched Gene Ontology (GO) terms for each pattern of *B. malayi* co-expressed transcripts calculated from a two-sided Fisher's exact test.

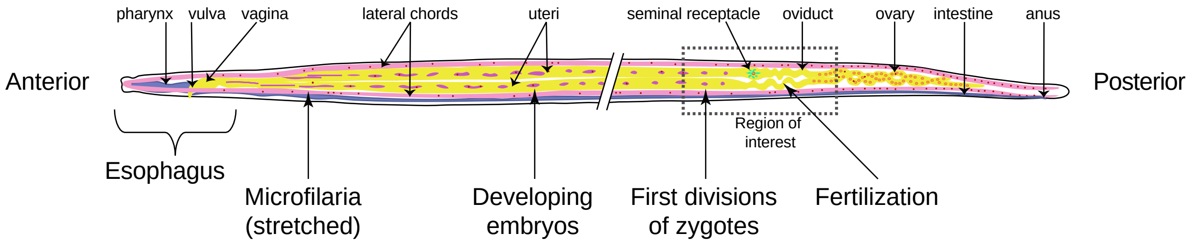

Supplementary Figure 3. Region of interest (ROI) used in the study. Schematic of the region of interest used in the study consisting of the posterior region of adult female *Brugia malayi* worms containing ovary tissue, the beginning of the uterus with fertilized eggs and early embryos, digestive tract, and body wall. Image adapted from [1].

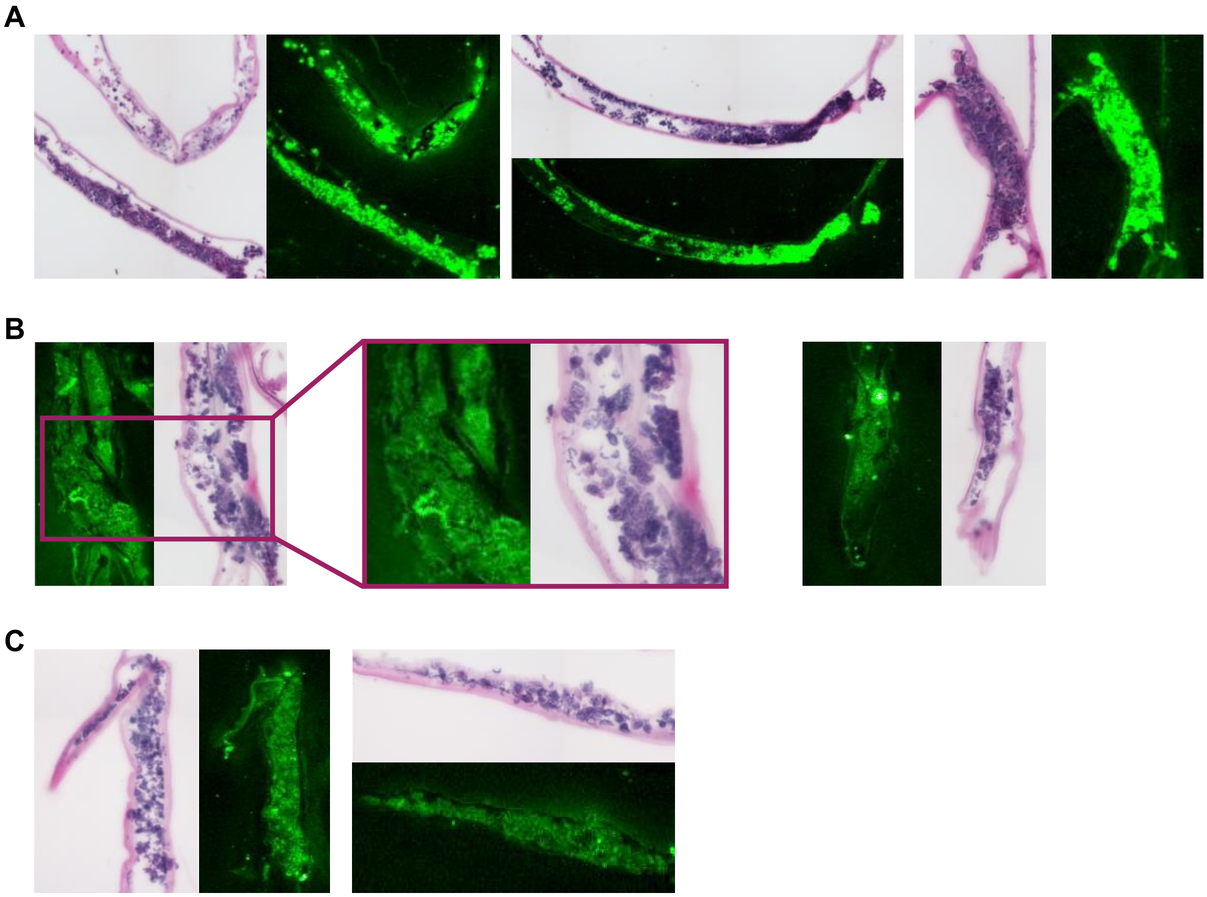

Supplementary Figure 4. Tissue optimization experiment images. Example images of the H&E image (left images) and corresponding Cy3 cDNA fluorescent image (right images) from tissue sections from whole adult *B. malayi* female worms that were permeabilized for 2 minutes (A), 2 minutes and 10 seconds (B), and 1 minute and 30 seconds (C).

Sample name	RIN	Description
OE_1	8	Total RNA from 2 worms from originally embedded blocks
OE_2	6	Total RNA from 3 worms from originally embedded blocks
RE_1	7	Total RNA from 2 worms from re-embedded blocks
RE_2	8	Total RNA from 3 worms from re-embedded blocks
D_OE_1	7	Total RNA from 2 doxycycline treated worms from originally embedded blocks
D_OE_2	6	Total RNA from 2 doxycycline treated worms from originally embedded blocks
D_RE_1	6	Total RNA from 2 doxycycline treated worms from re-embedded blocks
D_RE_2	6	Total RNA from 2 doxycycline treated worms from re-embedded blocks

Supplementary Table 1. RNA quality of total RNA from original and re-embedded worms from control and treated worms. Quality of total RNA, measured as RIN value, extracted from worms in the original OCT embedded blocks for control worms (OE_1 and OE_2) and doxycycline-treated worms (D_OE_1 and D_OE_2), and from the re-embedded blocks for control worms (RE_1 and RE_2) and doxycycline-treated worms (D_RE_1 and D_RE_2).

Supplementary Data

Supplementary Data 1. **Spatial transcriptomics summary.** Raw sequence library information for the samples and sample sections used in the study as output from 10X Genomics Space Ranger.

Supplementary Data 2. Clustering analysis of differentially expressed genes. Differentially expressed (DE) genes for each "miniatureST" cluster. Differential expression analysis used the Wilcoxon Rank Sum test and adjusted *p*-values (p_val_adj) were estimated with a two-sided alternative hypothesis. **A.** DE genes per cluster. **B.** DE genes corresponding to body wall (BW) markers in cluster 3. **C.** DE genes corresponding to reproductive tract (RT) markers in cluster 3. **D.** DE genes corresponding to digestive tract (DT) markers in cluster 1. **E.** DE genes corresponding to reproductive tract (RT) markers in cluster 2. **F.** DE genes corresponding to reproductive tract (RT) markers in cluster 4.

Supplementary Data 3. Fixed term enrichment analysis. Fixed term enrichment analysis results showing the genes and processes enriched in each cluster. Significantly overrepresented functional terms for each cluster were identified using a two-sided Fisher's exact test (FDR<0.05). **A.** All results for Fixed term enrichment analysis. **B.** Fixed term enrichment analysis results included in Figure 2E.

Supplementary Data 4. Pathways for genes of interest. Genes associated with glycolysis, gluconeogenesis, lactate dehydrogenase, and enzymes that convert cysteine amino acids to pyruvate.

Supplementary Data 5. Co-localization analysis of differentially expressed genes. Differentially expressed (DE) genes in *Wolbachia*+ versus *Wolbachia*- spots. Differential expression analysis used both the Wilcoxon Rank Sum test and DESeq2 test and adjusted *p*-values (p_val_adj) were estimated with a two-sided alternative hypothesis.

Supplementary Data 6. *B. malayi* genes correlating with *Wolbachia* abundance. Core *B. malayi* genes (membership ≥ 0.7) co-expressed across the different *Wolbachia* abundance groups (None, Low, Medium, High) in each pattern. **A.** Core genes (membership ≥ 0.7) co-expressed in Pattern 1. Gene Bm294 is WBGene00220555. **B.** Core genes (membership ≥ 0.7) co-expressed in Pattern 2. Gene Bm16 is WBGene00220277, Bma-bbs-8 is WBGene00226270, Bma-rpl-33.1 is WBGene00223531, Bm6111 is WBGene00226372, Bm13981 is WBGene00234242, Bm8720 is WBGene00228981. **C.** Core genes (membership ≥ 0.7) co-expressed in Pattern 3. Gene Bm17156 is WBGene00268299, Bma-enol-1 is WBGene00234226, Bma-hxk-1.1 is WBGene00233072, Bm9363 is WBGene00229624. **D.** Core genes (membership ≥ 0.7) co-expressed in Pattern 4.

Supplementary Data 7. Top Gene Ontology (GO) terms per pattern. Top Gene Ontology (GO) terms enriched in each pattern. Unadjusted *p*-values correspond to a two-sided Fisher's exact test. **A.** Top 20 enriched GO terms associated with the core genes (membership ≥ 0.7) of Pattern 1. **B.** Top 20 enriched GO terms associated with the core genes (membership ≥ 0.7) of Pattern 2. **C.** Top 20 enriched GO terms associated with the core genes (membership ≥ 0.7) of

Pattern 3. **D.** Top 20 enriched GO terms associated with the core genes (membership ≥ 0.7) of Pattern 4.

Supplementary Data 8. Spatial transcriptomics doxycycline-treated worms summary. Raw sequence library information for the doxycycline-treated samples and sample sections used in the study as output from 10X Genomics Space Ranger.

Supplementary Data 9. Gene expression changes in post-doxycycline treated worms. A. Average log base 2-fold changes and percentage of spots containing each gene in treated (pct.1) versus control (pct.2) spots. **B.** Average log base 2-fold changes and percentage of spots containing each gene in *Wolbachia*+ (pct.1) versus *Wolbachia*- (pct.2) spots.

References

1. Fischer K, Beatty WL, Jiang D, Weil GJ, Fischer PU. Tissue and Stage-Specific Distribution of Wolbachia in Brugia malayi. PLoS Negl Trop Dis [Internet]. Public Library of Science; 2011 [cited 2022 Sep 21];5:e1174. Available from: https://journals.plos.org/plosntds/article/file?id=10.1371/journal.pntd.0001174&type=printable
