# Supplementary material for: Miniature spatial transcriptomics for studying parasite-endosymbiont relationships at the micro scale
Source: Nat Commun. 2023 Oct 14;14:6500. doi: 10.1038/s41467-023-42237-y (PMC10576761; doi:10.1038/s41467-023-42237-y)
Supplement: Supplementary file 3 — Description of Additional Supplementary Files [file 41467_2023_42237_MOESM3_ESM.pdf]

Description of Additional Supplementary Files

File Name: **Supplementary Data 1**
Description: **Spatial transcriptomics summary.** Raw sequence library information for the samples and sample sections used in the study as output from 10X Genomics Space Ranger.

File Name: **Supplementary Data 2**
Description: **Clustering analysis of differentially expressed genes.** Differentially expressed (DE) genes for each "miniatureST" cluster. Differential expression analysis used the Wilcoxon Rank Sum test and adjusted p-values (p_val_adj) were estimated with a two-sided alternative hypothesis. **A.** DE genes per cluster. **B.** DE genes corresponding to body wall (BW) markers in cluster 3. **C.** DE genes corresponding to reproductive tract (RT) markers in cluster 3. **D.** DE genes corresponding to digestive tract (DT) markers in cluster 1. **E.** DE genes corresponding to reproductive tract (RT) markers in cluster 2. **F.** DE genes corresponding to reproductive tract (RT) markers in cluster 4.

File Name: **Supplementary Data 3**
Description: **Fixed term enrichment analysis.** Fixed term enrichment analysis results showing the genes and processes enriched in each cluster. Significantly overrepresented functional terms for each cluster were identified using a two-sided Fisher's exact test (FDR<0.05). **A.** All results for Fixed term enrichment analysis. **B.** Fixed term enrichment analysis results included in Figure 2E.

File Name: **Supplementary Data 4**
Description: **Pathways for genes of interest.** Genes associated with glycolysis, gluconeogenesis, lactate dehydrogenase, and enzymes that convert cysteine amino acids to pyruvate.

File Name: **Supplementary Data 5**
Description: **Co-localization analysis of differentially expressed genes.** Differentially expressed (DE) genes in *Wolbachia*+ versus *Wolbachia*- spots. Differential expression analysis used both the Wilcoxon Rank Sum test and DESeq2 test and adjusted pvalues (p_val_adj) were estimated with a two-sided alternative hypothesis.

File Name: **Supplementary Data 6**
Description: ***B. malayi* genes correlating with Wolbachia abundance.** Core *B. malayi* genes (membership ≥ 0.7) co-expressed across the different *Wolbachia* abundance groups (None, Low, Medium, High) in each pattern. **A.** Core genes (membership ≥ 0.7) co-expressed in Pattern 1. Gene Bm294 is WBGene00220555. **B.** Core genes (membership ≥ 0.7) co-expressed in Pattern 2. Gene Bm16 is WBGene00220277, Bma-bbs-8 is WBGene00226270, Bma-rpl-33.1 is WBGene00223531, Bm6111 is WBGene00226372, Bm13981 is WBGene00234242, Bm8720 is WBGene00228981. **C.** Core genes (membership ≥ 0.7) co-expressed in Pattern 3. Gene Bm17156 is WBGene00268299, Bma-enol-1 is WBGene00234226, Bma-hxk-1.1 is WBGene00233072, Bm9363 is WBGene00229624. **D.** Core genes (membership ≥ 0.7) coexpressed in Pattern 4.

File Name: **Supplementary Data 7**
Description: **Top Gene Ontology (GO) terms per pattern.** Top Gene Ontology (GO) terms enriched in each pattern. Unadjusted p-values correspond to a two-sided Fisher's exact test. **A.** Top 20 enriched GO terms associated with the core genes (membership ≥ 0.7) of Pattern 1. **B.** Top 20 enriched GO terms associated with the core genes (membership ≥ 0.7) of Pattern 2. **C.** Top 20 enriched GO terms associated with the core genes (membership ≥ 0.7) of Pattern 3. **D.** Top 20 enriched GO terms associated with the core genes (membership ≥ 0.7) of Pattern 4

File Name: **Supplementary Data 8**
Description: **Spatial transcriptomics doxycycline-treated worms summary.** Raw sequence library information for the doxycycline-treated samples and sample sections used in the study as output from 10X Genomics Space Ranger.

File Name: **Supplementary Data 9**
Description: **Gene expression changes in post-doxycycline treated worms. A.** Average log base 2-fold changes and percentage of spots containing each gene in treated (pct.1) versus control (pct.2) spots. **B.** Average log base 2-fold changes and percentage of spots containing each gene in *Wolbachia*+ (pct.1) versus *Wolbachia*- (pct.2) spots.
