# Supplementary material for: Miniature spatial transcriptomics for studying parasite-endosymbiont relationships at the micro scale
Source: Nat Commun. 2023 Oct 14;14:6500. doi: 10.1038/s41467-023-42237-y (PMC10576761; doi:10.1038/s41467-023-42237-y)
Supplement: Supplementary file 13 — Reporting Summary [file 41467_2023_42237_MOESM13_ESM.pdf]

nature portfolio

Corresponding author(s): Stefania Giacomello, Elodie Ghedin

Last updated by author(s): Sep 18, 2023

Reporting Summary

Nature Portfolio wishes to improve the reproducibility of the work that we publish. This form provides structure for consistency and transparency in reporting. For further information on Nature Portfolio policies, see our Editorial Policies and the Editorial Policy Checklist.

Statistics

For all statistical analyses, confirm that the following items are present in the figure legend, table legend, main text, or Methods section.

n/a	Confirmed	
☐	☒	The exact sample size (*n*) for each experimental group/condition, given as a discrete number and unit of measurement
☐	☒	A statement on whether measurements were taken from distinct samples or whether the same sample was measured repeatedly
☐	☒	The statistical test(s) used AND whether they are one- or two-sided<br>*Only common tests should be described solely by name; describe more complex techniques in the Methods section.*
☐	☒	A description of all covariates tested
☐	☒	A description of any assumptions or corrections, such as tests of normality and adjustment for multiple comparisons
☒	☐	A full description of the statistical parameters including central tendency (e.g. means) or other basic estimates (e.g. regression coefficient) AND variation (e.g. standard deviation) or associated estimates of uncertainty (e.g. confidence intervals)
☐	☒	For null hypothesis testing, the test statistic (e.g. *F*, *t*, *r*) with confidence intervals, effect sizes, degrees of freedom and *P* value noted<br>*Give P values as exact values whenever suitable.*
☒	☐	For Bayesian analysis, information on the choice of priors and Markov chain Monte Carlo settings
☐	☒	For hierarchical and complex designs, identification of the appropriate level for tests and full reporting of outcomes
☐	☒	Estimates of effect sizes (e.g. Cohen's *d*, Pearson's *r*), indicating how they were calculated

Our web collection on statistics for biologists contains articles on many of the points above.

Software and code

Policy information about availability of computer code

Data collection	The spatial transcriptomics sequence data was generated using Visium Spatial Gene Expression assay (10X Genomics) kits and sequenced on a Illumina NextSeq 2000 with paired-end, dual index sequencing, at a depth of 9.9-23.8 million reads per sample. Read 1 was sequenced for 28 cycles and Read 2 was sequenced with 150 cycles. A Zeiss Axiolmager.Z2 VSlide Microscope using the Metasystems VSlide scanning system with Metafer 5 v3.14.179 and VSlide software was used to acquire all tissue images.
Data analysis	Read 2 adapter sequences were trimmed using cutadapt (v2.3) with a custom bash script (https://github.com/ludvigla/VisiumTrim). 10X Genomics Loupe Browser (v4.0.0) was used to manually select spots under tissue sections in the H&E jpeg images. Genomic sequence and annotation files for Brugia malayi were acquired from WormBase: WBPS14 and for Wolbachia were acquired as RefSeq: NC_006833.1. gffread from cufflinks (v2.2.1) was used to convert the Brugia malayi annotation file from GFF to GTF. 10X Genomics Space Ranger (v1.2.0) was used to build a combined Brugia malayi-Wolba chia reference. Gene expression matrices were generated with 10X Genomics Space Ranger (10X Genomics Space Ranger (v1.2.0). Count matrices were analyzed in R (v4.0.3 and v4.0.5) using STUtility (v1.0) (https://github.com/jbergenstrahle/STUtility) and Harmony (v0.1.0) (https://github.com/immunogenomics/harmony). 3D model and shiny app generation was done in R (v4.2.0) using the STUtility package (v1.0). MFuzz69 (v2.50.0) was used to perform soft clustering of B. malayi genes across Wolbachia abundance and biomaRt (v2.46.3) and topGO70 (v2.42.0) for running GO term enrichment analysis of the resulting core pattern genes in R (v4.0.3). The scripts used to generate count matrices from raw sequence fastq files and related R scripts used to analyze count matrices for quality control and filtering, normalization, clustering and differential expression analysis, colocalization analysis, 3D figure generation, and treated worm analysis can be accessed from our github repository (https://github.com/giacomellolab/Brugia_malayi_study).

For manuscripts utilizing custom algorithms or software that are central to the research but not yet described in published literature, software must be made available to editors and reviewers. We strongly encourage code deposition in a community repository (e.g. GitHub). See the Nature Portfolio guidelines for submitting code & software for further information.

Data

Policy information about availability of data

All manuscripts must include a data availability statement. This statement should provide the following information, where applicable:

- Accession codes, unique identifiers, or web links for publicly available datasets
- A description of any restrictions on data availability
- For clinical datasets or third party data, please ensure that the statement adheres to our policy

Raw sample sequence fastq files are available on NCBI SRA under the accession PRJNA870734 (http://dx.doi.org/https://www.ncbi.nlm.nih.gov/sra/PRJNA870734). Processed gene count matrices, related metadata, and corresponding ST tissue H&E microscopy images, and 3D model HTML files are available in the Mendeley dataset under Reserved DOI: 10.17632/8f62vydg3z.1. The gene expression information for all the Brugia malayi genes from the normalized SCT assay for the control worms are available for visualization on our publicly available app: https://giacomellolabst.shinyapps.io/brugiast-shiny/. B.malayi gene annotations and identified orthologs in C. elegans were obtained from wormbase.org. WormBase ParaSite74 (WBPS18) [https://parasite.wormbase.org/] was used to get Gene Ontology (GO) Terms. Brugia malayi genome assembly PRJNA10729 WormBase release WBPS14 was used (https://ftp.ebi.ac.uk/pub/databases/wormbase/parasite/releases/WBPS14/species/brugia_malayi/PRJNA10729/). Wolbachia genome assembly were acquired as RefSeq: NC_006833.1 (https://www.ncbi.nlm.nih.gov/datasets/genome/GCF_000008385.1/).

Human research participants

Policy information about studies involving human research participants and Sex and Gender in Research.

Reporting on sex and gender	N/A
Population characteristics	N/A
Recruitment	N/A
Ethics oversight	N/A

Note that full information on the approval of the study protocol must also be provided in the manuscript.

Field-specific reporting

Please select the one below that is the best fit for your research. If you are not sure, read the appropriate sections before making your selection.

☒ Life sciences ☐ Behavioural & social sciences ☐ Ecological, evolutionary & environmental sciences

For a reference copy of the document with all sections, see nature.com/documents/nr-reporting-summary-flat.pdf

Life sciences study design

All studies must disclose on these points even when the disclosure is negative.

Sample size	No sample size calculation was performed. Sample size was chosen based on sample availability. Several tissue sections were placed on the same Visium capture array to maximize sample size. 30 tissue sections were collected across 3 biological replicates of Brugia malayi adult female control worms with 9 sections on one capture array for sample BM1, 11 section on one capture area for sample BM2, and 10 sections on one capture array for sample BM3. 5 tissue sections were collected across 2 biological replicates of Brugia malayi adult female doxycycline treated worms and placed across 2 capture arrays. We deemed we had sufficient data as we were able to demonstrate high reproducibility of gene expression information captured across and within samples and were able to explore gene expression patterns and obtain meaningful results with methods such as non-supervised clustering with this amount of data.
Data exclusions	No data was excluded from the analysis.
Replication	The spatial transcriptomics data presented in the manuscript main figures and supplementary information was collected from 3 biological replicates of Brugia malayi adult female control worms (9 section from sample BM1, 11 sections from sample BM2, 10 sections from sample BM3) and 2 biological replicates of Brugia malayi adult female doxycycline treated worms (5 tissue sections total) from the same collection batch (the attempts at replication were successful). The data presented in Supplementary Table 2 is from total RNA extraction experiments done on n=2 replicates (tissue sections extracted in bulk from n=2 worms for one replicate and n=3 worms for the second replicate) from originally embedded tissue blocks, n=2 replicates (tissue sections extracted in bulk from n=2 worms for one replicate and n=3 worms for the second replicate) from re-embedded tissue blocks, n=2 replicates (tissue sections extracted in bulk from n=2 worms for one replicate and n=3 worms for the second replicate) from originally embedded tissue blocks from treated worms, and n=2 replicates (tissue sections extracted in bulk from n=2 worms for one replicate and n=3 worms for the second replicate) from re-embedded tissue blocks of treated worms (the attempts at replication were successful).
Randomization	All worm samples were processed in the same batch. Covariates were not controlled because they were not relevant to the study as there

Randomization	were only a small number of worm samples used in this study.
Blinding	The results were not performed blindly since they did not include subjective measurements and were performed by individual researchers.

Reporting for specific materials, systems and methods

We require information from authors about some types of materials, experimental systems and methods used in many studies. Here, indicate whether each material, system or method listed is relevant to your study. If you are not sure if a list item applies to your research, read the appropriate section before selecting a response.

Materials & experimental systems

n/a	Involved in the study
☒	☐ Antibodies
☒	☐ Eukaryotic cell lines
☒	☐ Palaeontology and archaeology
☐	☒ Animals and other organisms
☒	☐ Clinical data
☒	☐ Dual use research of concern

Methods

n/a	Involved in the study
☒	☐ ChIP-seq
☒	☐ Flow cytometry
☒	☐ MRI-based neuroimaging

Animals and other research organisms

Policy information about studies involving animals; ARRIVE guidelines recommended for reporting animal research, and Sex and Gender in Research

Laboratory animals	Adult Brugia malayi female parasitic worms (>120 dpi) were recovered from the peritoneal cavity of infected gerbils (Meriones unguiculatus) and were obtained from FR3, University of Georgia, Athens.
Wild animals	No wild animals were collected for use in this study.
Reporting on sex	As we were interested in studying tissues relevant for oogenesis and embryogenesis, processes that only occur in adult female Brugia malayi worms, we chose adult female Brugia malayi worms for our study. The findings of the study apply to adult female Brugia malayi worms.
Field-collected samples	No field collected animals were used in the study.
Ethics oversight	All animal work conducted by the NIH/NIAID Filariasis Research Reagent Resource Center (FR3) followed the national and international guidelines outlined by the National Institutes of Health Office of Laboratory Animal Welfare and was approved by the University of Georgia Athens.

Note that full information on the approval of the study protocol must also be provided in the manuscript.
